# Factors influencing malignant mesothelioma survival: a retrospective review of the National Mesothelioma Virtual Bank cohort

**DOI:** 10.12688/f1000research.15512.3

**Published:** 2019-06-03

**Authors:** Waqas Amin, Faina Linkov, Douglas P. Landsittel, Jonathan C. Silverstein, Wiam Bshara, Carmelo Gaudioso, Michael D. Feldman, Harvey I. Pass, Jonathan Melamed, Joseph S. Friedberg, Michael J. Becich

**Affiliations:** 1 Biomedical Informatics, University of Pittsburgh, Pittsburgh, PA, 15206, USA; 2 Obstetrics, Gynecology and Reproductive Science,, University of Pittsburgh, Pittsburgh, PA, 15213, USA; 3 Department of Pathology and Lab. Medicine, Roswell Park Comprehensive Cancer Center, Buffalo, NY, 14263, USA; 4 Department of Biostatistics & Bioinformatics, Roswell Park Cancer Institute, Buffalo, NY, 14263, USA; 5 Department of Pathology and Laboratory Medicine, The Hospital of the University of Pennsylvania, Perelman School of Medicine, Philadelphia, PA, 19104, USA; 6 Department of Surgery, New York University Langone Health, New York, NY, 10016, USA; 7 Department of Pathology, New York University Langone Health, New York, NY, 10016, USA; 8 Department of Surgery, University of Maryland School of Medicine, Baltimore, MD, 21201, USA

**Keywords:** Mesothelioma, Survival analysis. Cox hazard regression analysis, Biobanking, Risk factor

## Abstract

**Background**: Malignant mesothelioma (MM) is a rare but deadly malignancy with about 3,000 new cases being diagnosed each year in the US.  Very few studies have been performed to analyze factors associated with mesothelioma survival, especially for peritoneal presentation. The overarching aim of this study is to examine survival of the cohort of patients with malignant mesothelioma enrolled in the National Mesothelioma Virtual Bank (NMVB).

**Methods: ** 888 cases of pleural and peritoneal mesothelioma cases were selected from the NMVB database, which houses data and associated biospecimens for over 1400 cases that were diagnosed from 1990 to 2017. Kaplan Meier’s method was performed for survival analysis. The association between prognostic factors and survival was estimated using Cox Hazard Regression method and using R software for analysis.

**Results: **The median overall survival (OS) rate of all MM patients, including pleural and peritoneal mesothelioma cases is 15 months (14 months for pleural and 31 months for peritoneal).  Significant prognostic factors associated with improved survival of malignant mesothelioma cases in this NMVB cohort were younger than 45, female gender, epithelioid histological subtype, stage I, peritoneal occurrence, and having combination treatment of surgical therapy with chemotherapy. Combined surgical and chemotherapy treatment was associated with improved survival of 23 months in comparison to single line therapies.

**Conclusions:** There has not been improvement in the overall survival for patients with malignant mesothelioma over many years with current available treatment options. Our findings show that combined surgical and chemotherapy treatment in peritoneal mesothelioma is associated with improved survival compared to local therapy alone.

## Introduction

Malignant mesothelioma is a rare and fatal malignancy, associated with occupational and environmental exposure to asbestos. As per American Cancer Society, approximately 3000 new cases are diagnosed per year in the United States. The pleura is the primary site of mesothelioma occurrence, but it also occurs at other sites (pericardium, peritoneum, tunica vaginalis testis) ^1, 2^. For pleural mesothelioma, the median overall survival age ranges from 21 months (for Stage I) to 12 month (for Stage IV) disease ^3^. In the 1970s, the incidence of mesothelioma cases started to increase, and it became evident that the occupational and environmental exposures to asbestos (occurring during 1930s–1970s) were associated with the increased incidence of this fatal disease ^4^. Despite regulations aimed to ban the industrial use of asbestos by US Occupational Safety and Health Administration (OSHA) in 1970, data do not suggest a decline in the incidence of malignant mesothelioma in the U.S. ^5^. However, the impact of these changes are difficult to assess due to the fact that mesothelioma is typically diagnosed decades after the initial asbestos exposure ^6^. A recent multisite cohort investigation reported that the median time of diagnosis from the first environmental exposure was 38.4 years (IQR 31.3–45.4 years) ^7^. Both genetics and environmental exposure plays a critical role in acquiring malignant mesothelioma. BAP1 is the only gene reported to be in a causal pathway for malignant mesothelioma development in connection with asbestos exposure. BAP1 germ line mutation has been found to be a risk factor for the development of malignant mesothelioma in families where the mutation is found in 50% of members. This mutation has also found to be linked to the development of BAP1 cancer syndrome, characterized by an increased incidence of malignant mesothelioma, uveal and cutaneous melanoma, and melanocytic BAP1-mutated atypical intradermal tumors ^8, 9^.

The majority of pleural malignant mesothelioma cases in men and women are linked to exposure to asbestos. Asbestos particle exposure can occur from indoor and outdoor commercial and naturally occurring asbestos. Naturally Occurring Asbestos (NOA) concentration in soil is commonly below the threshold for detection by light microscopy but nevertheless can still cause potential hazardous airborne exposure. Detection of environmental exposure to NOA is much more challenging than detection of commercial exposure to asbestos. Activity based sampling is considered to be very important for health risk assessment and to characterize the environmental exposure ^10, 11^. Another approach is to study high risk populations and screen patients with benign pleural disease through radiographic imaging. Previous research has also explored the differences between the development of malignant mesothelioma in patients that had environmental exposure like NOA, and those that have genetic risk factors in conjunction with occupational exposure (even at a low level). In cases of malignant mesothelioma that have been linked to environmental exposure, BAP1 mutations have mainly been seen in younger population with equal gender and pleural/peritoneal distribution ^12, 13^.

After pleura, the peritoneum is the second most frequent site of origin of mesothelioma ^14^. Epidemiological studies of peritoneal mesothelioma are limited by the rarity of this disease, as well as by possible geographic and temporal variations in diagnostic practice ^15^. While survival for patients with peritoneal mesothelioma is more favorable, with patients surviving up to 60 months ^16, 17^, limited number of studies have explored factors affecting the survival of peritoneal mesothelioma.

However, given the rarity of the disease, few databases have sufficient number of cases and treatment data to make analysis of therapeutic options with statistical significance possible. NMVB is an especially valuable resource for mesothelioma research, as beyond its capability as a biorepository it includes well annotated data for populations residing in Pennsylvania and New York states (two of the top 5 states for mesothelioma-associated mortality) ^18^. Previous SEER (Surveillance, Epidemiology and End Result Program) based studies exploring factors that influence mesothelioma have not included populations residing in Pennsylvania and New York ^19^.

Previously published research of pleural mesothelioma suggest that histological type (epithelioid) and early stages are associated with improved survival following surgical treatment ^20^. Other predictive factors explored in previously published literature including gender, advanced age, weight loss, chest pain, poor performance status, as well as low hemoglobin, leukocytosis, and thrombocytosis. It has been suggested that female patients with mesothelioma have a better life expectancy as compared to male patients ^21^.

Currently there are few therapeutic options, including surgery, chemotherapy, radiation therapy and a combination of these options that may significantly improve the overall survival from this deadly disease ^22^. Considering the aggressive nature and poor prognosis associated with this disease, improving our existing knowledge regarding the biology of the disease and factors predictive of the efficacy of existing therapeutic options and treatment regiments for malignant mesothelioma is critical.

In this study, we analyzed malignant mesothelioma cases from the National Mesothelioma Virtual Bank (NMVB) to evaluate the effect of clinical, pathological, and epidemiological factors, and therapeutic options as determinants of overall survival. Thus our study adds geographic breadth to the existing mesothelioma research knowledge. Additionally, our dataset includes cases of peritoneal mesothelioma, which were not the focus of previous studies.

## Methods

### Ethical considerations

This study is conducted under the Institutional Review Board (IRB) approval (IRB #0608194) of NMVB and its supporting sites, with approval from the principal investigator of NMVB to use the de-identified data from the resource.

### Data source

The patient cohort for this study (n=888) is selected from the NMVB resource, which contains data and biospecimens from both pleural and peritoneal malignant mesothelioma cases. The NMVB database records treatment type in general categories of “cancer directed surgery alone, surgery combined with chemotherapy, as well as surgery combined with chemotherapy and radiation”. The specific details of treatment (such as exact surgery type of type of chemotherapy regimen used) are not recorded in the NMVB. The NMVB enrolls patients from NMVB collaborating sites (New York University, University of Pennsylvania, University of Maryland, Roswell Park Cancer Institute and University of Pittsburgh Medical Center), located in the north east region of the USA. This geographic emphasis has the potential for a selection bias as few patients are enrolled from the other regions of the country due to NMVB network coverage. NMVB was developed to collect mesothelioma biospecimens and data from prospectively consented as well as retrospectively identified patients, which allows for capture of both previous and currently treated cases of mesothelioma.

### Patient selection

**Figure 1.**
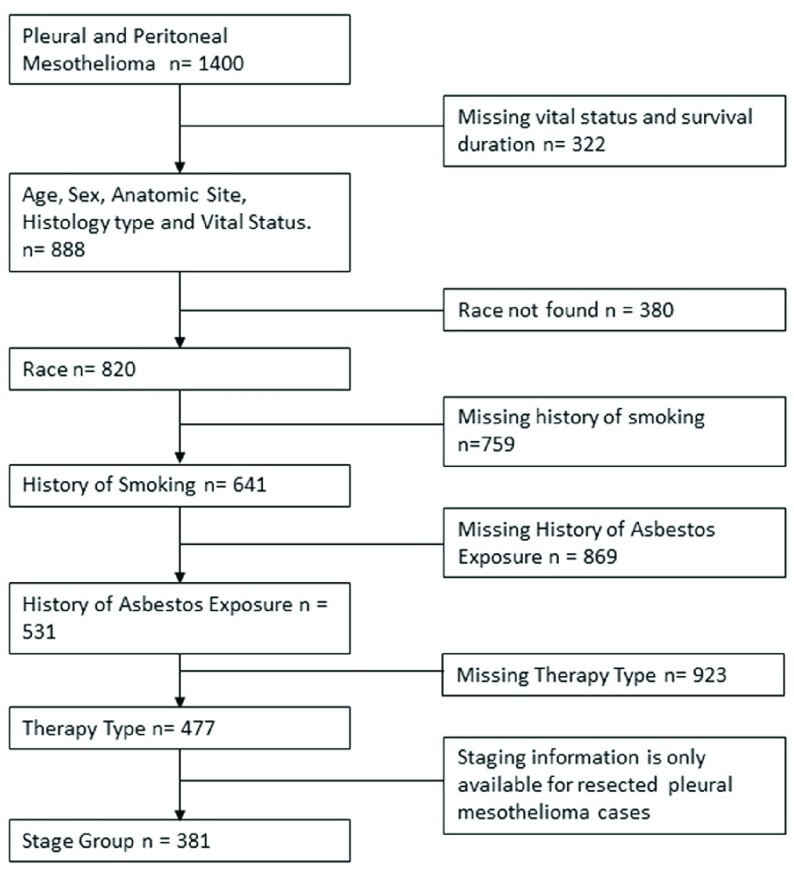
Study workflow and case inclusion and exclusion criteria.

Demographic, treatment, clinical and survival information of histologically confirmed pleural and peritoneal mesothelioma patients diagnosed between 1999 and 2017 were obtained from the NMVB database. Inclusion criteria included the following: confirmed diagnosis of malignant mesothelioma (limited to pleural and peritoneal presentation), availability of complete data on age, gender, race, asbestos exposure, smoking history, history of alcohol use, histological type, site of tumor, disease stage (for pleural presentation), vital status, and survival duration. Exclusion criteria included the following: benign mesothelioma, and tumor site other than pleura and peritoneum. This investigation was limited to the most common histological subtypes of diffuse malignant mesothelioma including biphasic, epithelial or epithelioid, and sarcomatoid. The desmoplastic histology subtype is classified as sarcomatoid, and papillary mesothelioma as epithelial or epithelioid ^23, 24^. For the purpose of this study, tumor anatomic site is classified into two main categories: pleura (which includes visceral/parietal pleura and lung, chest wall, ribs) and peritoneum (includes peritoneal cavity and organs involved). This analysis focused on 888 participants that met the inclusion criteria. Patient characteristics are presented in Table 1. Case selection flow is presented in Figure 1.

**Table 1.** Patient characteristics.

Variables	Number of patients
Age	888
18–44	49
45–54	102
55–64	266
65–74	312
75 +	161
Gender	888
Male	683
Female	205
Anatomic Site	888
Pleural	740
Peritoneum	148
Histology	888
Epithelial or epithelioid	636
Biphasic	165
Sarcomatoid	87
Race	820
European American	792
Non-European American	28
History of Smoking	641
Yes	364
No	277
History of Asbestos Exposure	531
Yes	413
No	118
Stage Group (limited to pleural cases)	381
I	178
II	24
III	157
IV	22
Therapy Type	477
Surgery	101
Surgery + Chemo	327
Surgery + Chemo + Radiation	49

### Definition of staging and metastatic disease

We have performed analysis of staging data for pleural mesothelioma cases that have undergone surgical resection, however used a surrogate staging system for peritoneal mesothelioma as there is no formal TNM staging system for peritoneal malignant mesothelioma. We converted the TNM staging of pleural mesothelioma into stage grouping as per College of American Pathology (CAP) protocol 2017 for pleural malignant mesothelioma. Metastatic disease status was defined as the tumor spread from the point of origin to the lymph node and other organs in the body.

### Statistical analyses

We included the following variables in the analysis: age, gender, race, smoking history, history of alcohol, asbestos exposure, site of tumor, histological type, treatment, staging and outcome variables including vital status and survival period. Duration of observation was defined as time (in months) between date of initial diagnosis until death (vital status = expired) or the date of last known contact for each participant. Smoking history was analyzed as a dichotomous variable (yes/no), where current, past and smoking for a brief period of time, were grouped as positive history of smoking (yes). The contribution of the three treatment types on mesothelioma survival rate is evaluated in this study.

We constructed survival curves using the Kaplan-Meier method for the entire dataset, followed by a separate analysis limited to female patients. We also performed a separate Kaplan-Meier analysis for peritoneal cases only. We performed Log-rank test of equality across strata for categorical variables. We analyzed the independent contribution to mesothelioma survival of several prognostics with univariable and multivariable regression methods based on the Cox proportional hazards model. Variables were entered into the model using a forward selection approach, starting with the most significant variable (based on the unadjusted p-value) and then continuing in order of significance. We analyzed factors contributing to mesothelioma survival separately for cases with complete data and with missing data to rule out any systematic bias associated with cases with missing data. Two-tailed p-values less than 0.05 were considered significant. We used The R Project (version 3.4.0) for Statistical Computing to perform all analysis ^25^.

## Results

The majority of patients were European American (97%) and male (77%). History of smoking was reported by 364 (57 %) patients among n=641 and history of asbestos exposure was reported in 413 cases (78 %) among n= 531. Epithelial or epithelioid histological subtype was the most prevalent histology in 71.4% of cases in this dataset (n = 636). Cancer directed surgery was performed in 54 % cases, while surgery and chemotherapy treatment jointly was administered in 37% of cases. The median overall survival of the cohort was 15 months. Table 2 and Figure 3 demonstrate the results of the univariable and multivariable analysis respectively (Cox proportional hazard regression models).

**Table 2.** Unadjusted Cox Hazard Regression Analysis, predictors of mesothelioma survival (n=888). Ref – Reference group.

Variable	Hazard ratio	95% Confidence interval p value for trend
**Age**		
18–44	1.00	Ref
45–54	2.0	1.3-3 P=0.001
55–64	2.3	1.6-3.3 P<0.001
65–74	2.7	1.8-3.9 P<0.001
75+	3.4	2.3-5.1 P<0.001
**Gender**		
Female	1.0	Ref
Male	1.6	1.4.0-1.9 P<0.001
**Anatomic site**		
Peritoneum	1.0	Ref
Pleural	2.1	1.7-2.6 P<0.001
**Therapy**		
Surgery	1.0	Ref
Surgery, chemo	0.49	0.39-0.62 P<0.001
Surgery, chemo, radiation	0.63	0.44-0.90 P=0.011
**Smoking history**		
No	1.0	Ref
Yes	1.2	1-1.5 P=0.022
**Stage (pleural cases only)**		
I	1.0	Ref
II	1.3	0.82-2.0 P<0.27
III	1.7	1.31-2.1 P<0.001
IV	2.0	1.24-3.2 P=0.004
**Histology**		
Biphasic	1.0	Ref
Epithelial or epithelioid	0.48	0.40-0.57 P<0.001
Sarcomatoid	0.97	0.74-1.26 P=0.797
**Race**		
Non European American	1.0	Ref
European American	1.8	1.1-2.8 P<0.012
**Asbestos Exposure**		
Yes	1.0	Ref
No	0.61	0.48-0.78 P<0.001

**Figure 2.**
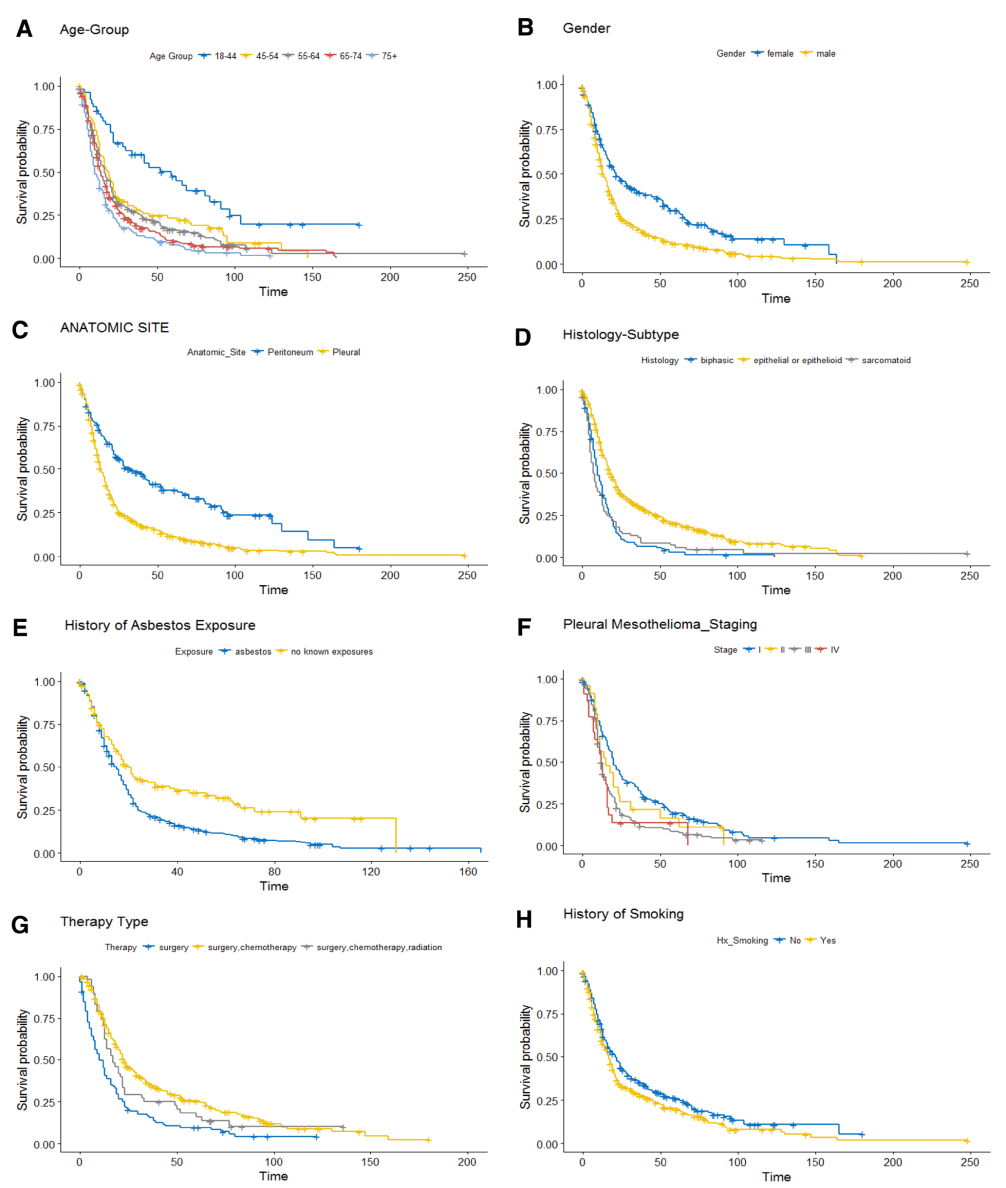
Kaplan Meier Curve analysis performed at age ( **a**), gender ( **b**), anatomic site ( **c**), histology subtype ( **d**), history of asbestoses exposure ( **e**), staging (pleural mesothelioma) ( **f**), therapy type ( **g**), and history of smoking ( **h**).

Overall, the non-parametric univariate Kaplan Meier analysis and log rank tests demonstrated longer survival in younger age group (18–44 years), female gender, with no known asbestos exposure history, epithelioid histological type, combined surgical and chemotherapy, Stage I, or peritoneum presentation ( Figure 2a–2i).

The median survival for age group 18–44 years was 59 months (95% CI: 34 - 91) but much less favorable for the age group 75 and over, at 10 months (95% CI: 9 – 13). The median survival for females was 22 months (95% CI: 18 - 30) as compared to 14 months for males (95% CI: 13-16). The group with no reported history of asbestos exposure had a median survival rate of 20 months (95% CI: 16 - 31), as compared to median survival of 15 months (95% CI: 13-17) for the group with reported exposure. The epithelioid histological type median had a median survival of 18 months (95% CI: 17-21) as compared to 10 months for biphasic (95% CI: 9-13) and 7 months for sarcomatoid subtype (95%CI: 6-11). The European American group had a median survival of 15 months (95% CI: 13 – 16) as compare to median survival of 34 months (95% CI: 21-83) in non-European American population. The analysis suggests patients receiving combined therapies [(surgical and chemotherapy (95% CI: 13-19), surgical plus chemotherapy and radiation therapy (95% CI: 10-21)] had a more favorable median survival period in comparison to those with single line surgical therapy (95% CI: 8-14). Overall, median OS was most favorable (23 months (95% CI 21 to 27 months)) for patients treated with combined surgery and chemotherapy. Adding radiation to chemotherapy did not improve survival.

The median survival period for stage I group (including stages IA and IB) was 20 months (95% CI: 18 – 25) as compared to 12 months for stages III and IV. Presentation in the peritoneum site and no history of smoking was also associated with improved survival ( Figure 1). When stratified by anatomic site of tumor, the median survival period among patients with peritoneal mesothelioma, who received surgical and chemotherapy, demonstrated longer survival of 28 months (95% CI: 28 – 45) as compared to 14 months (95% CI: 11 – 17) in patients with pleural mesothelioma.

Overall, multivariable analysis confirmed that younger age groups, female gender, peritoneal anatomic site, combination of surgery and chemotherapy, no history of smoking, early stage (I and II), and epithelial histology were all predictors of more favorable survival ( Table 2).

**Figure 3.**
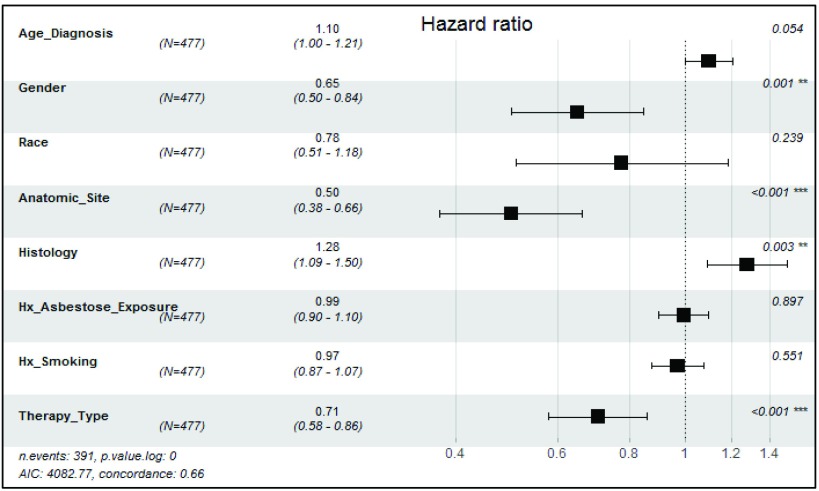
Adjusted Cox Hazard analysis, predictors of mesothelioma survival, multivariable analysis (n=477).

In addition, we performed multivariable cox hazard proportional analysis on the complete dataset of n= 477 which had no missing record variables that has obtained from the primary dataset (n= 888). We included all the predictive prognostic variables except for stage, because there is no established TNM staging for peritoneal mesothelioma. We presented these results as supplementary analysis in Figure 3.

## Discussion and conclusion

The focus of this study has been on the exploration of risk factors affecting mortality in the states of Pennsylvania and New York, which represent a region with an aging population, environmental concerns, well documented history of asbestos exposure, and other risk factors associated with mesothelioma development. This region has not been comprehensively covered in previously reported investigations. In addition to expanding the geographic region in this study, another added value of this study is that we explored factors contributing to survival for peritoneal mesothelioma separately from those for the more prevalent pleural mesothelioma. **S**urvival analysis on the NMVB cohort demonstrated that patient age younger than 45, female gender, epithelioid histological subtype, Stage I of the disease, peritoneum as primary site and surgical therapy combined with chemotherapy were favorable prognostic factors. This study corroborates the analysis of the SEER data by Taioli *et al.* suggesting that female gender, younger age, early stage, and surgery alone are all good prognostic factors ^19^. This study also corroborates previous investigations suggesting that peritoneal presentation, especially among women, is associated with longer survival ^26^.

Consistent with the literature, our data suggests that women have longer survival in comparison to men, which may be due to factors like lower levels of smoking amongst females and/or different levels of environmental exposure ^21, 27– 30^. Specifically, women may be more likely to have para-occupational exposures, which typically refer to an asbestos-exposed worker serving as a vector for the transport of fibers to the household setting and family members. Other terms used in this context include household contact, take-home exposure or domestic exposure ^31^. Exact factors explaining survival advantage among women needs to be further investigated in future research.

Strengths of this study include the use of a very large dataset collected utilizing uniform data collection protocol. The weaknesses of this study include lack of detailed data on specific surgical treatment type and also the fact that exposure data is self-reported and not corroborated by radiologic analyses. We also recognize that our population may not be representative of the entire population of mesothelioma patients, as large number of patients in the general population are not good surgical candidates. Additionally, while we attempted to obtain detailed occupational exposure data for asbestos and other substances, participants’ ability to recall the duration and details of their exposure is a potential source of bias. In our future investigations, we will also focus on BAP1 mutations. We will also focus our investigation on patients who are not surgical candidates, including from ethnic minorities, and younger patients.

Malignant mesothelioma is a life-threatening condition that has been under-investigated and warrants greater investigation, considering that it is a lethal disease associated with high mortality with short survival and its incidence has not shown signs of improvement over the past several decades. Further studies are needed to evaluate screening, diagnostic, staging and treatment for various subtypes of mesothelioma.

In thefuture, it would be particularly interesting to include in the cohort cases that do not qualify for surgical management because of advanced disease. An improved understanding of factors associated with mesothelioma morbidity and mortality may help identify high-risk groups based on different occupational exposures. Such groups may be further evaluated for responsiveness to innovative management strategies for mesothelioma. The identification of these factors could help stratify patients at risk for therapy failure who may benefit from novel interventions or could avoid treatments that are not effective or with high mortality risk. We hope our report underscored the significant value of NMVB as a national research resource pairing data and biospecimens which are made available (through an application process) to the entire research community. We envision that in the future, existing information and biospecimen repositories like NMVB will be harnessed to greater extent and foster greater investigation studies into rare diseases like mesothelioma.

## Data availability

An investigator can obtain de-identified data from National Mesothelioma Virtual Bank by application process: 1) submit a letter of intent (LOI) ( https://mesotissue.org/node/26) to the NMVB (email address). The NMVB Research Evaluation Panel (REP), composed of extramural scientists with varied expertise including laboratory science, lung pathology, mesothelioma, and statistics ( https://mesotissue.org/rep) then reviews requests for scientific merit and provides recommendations for approval. Thereafter once a data (or material in case of request for biospecimen) use agreement (DUA) has been concluded between investigator and NMVB, the data (or biospecimen) can be provided to the applicant.
