# Supplementary material for: Factors influencing malignant mesothelioma survival: a retrospective review of the National Mesothelioma Virtual Bank cohort
Source: F1000Res. 2019 Jun 3;7:1184. Originally published 2018 Aug 3. [Version 3] doi: 10.12688/f1000research.15512.3 (PMC6198263; doi:10.12688/f1000research.15512.3)
Supplement: Peer review history

# Peer review: Reviewer response for version 2

**DOI:** 10.5256/f1000research.19194.r42130

**Published:** 2019-01-15

**Authors:** Nico van Zandwijk

**Affiliations:** 1 University of Sydney, Sydney, NSW, Australia; 2 Sydney Local Health District, Sydney, NSW, Australia

I agree with Dr Amin that this a very valuable study, describing 1% of national (USA) mesothelioma cases, that were not part of the SEER database. In the revised version the outcomes of this prognostic factors study have been discussed in detail, while the limitations of the dataset were clearly addressed. The results from previous prognostic factors studies are mostly confirmed. The prognostic data of patients with peritoneal mesothelioma, who received combined modality treatment, are considered especially important and may well assist in drafting guidelines for this selected group of patients.

Is the work clearly and accurately presented and does it cite the current literature?

Yes

If applicable, is the statistical analysis and its interpretation appropriate?

I cannot comment. A qualified statistician is required.

Are all the source data underlying the results available to ensure full reproducibility?

Yes

Is the study design appropriate and is the work technically sound?

Partly

Are the conclusions drawn adequately supported by the results?

Partly

Are sufficient details of methods and analysis provided to allow replication by others?

Yes

Reviewer Expertise:

NA

I confirm that I have read this submission and believe that I have an appropriate level of expertise to confirm that it is of an acceptable scientific standard.

# Peer review: Reviewer response for version 1

**DOI:** 10.5256/f1000research.16914.r39375

**Published:** 2018-10-19

**Authors:** Tobias Peikert

**Affiliations:** 1 Division of Pulmonary and Critical Care Medicine, Mayo Clinic, Rochester, MN, USA

This manuscript summarizes the data from a large cohort of patients with pleural and peritoneal mesothelioma from the National Mesothelioma Virtual Tissue Bank (NMVB). The cases are from New York and Pennsylvania. The study confirms findings from prior analysis of the SEER database, which did not include patients from these states. Patient survival is dependent on age, gender, disease stage, disease site (pleural versus peritoneal), histological subtype and presence of multi-modality therapy. However there are several limitations.

1. As with most large mesothelioma databases surgically treated patients are over represented. In fact only 10-15% of patients with mesothelioma are candidates for surgery and most patients are treated with systemic chemotherapy. Consequently despite being a large cohort the study population is not representative of the majority of mesothelioma patients. Future studies should include a representative proportion of non-surgical patients.
2. The conclusions about differences in therapy are difficult to interpret since not detailed treatment data (curative versus palliative), R1 versus >R1 resection, type of surgery and type of radiation therapy are not collected. It is also not clear of the patients in fact completed all therapies listed. In addition treatment data was only available in a subset of patients.
3. In regards to the younger age patients BAP1 mutation status would be very helpful to explore. Some of this data should be available since the NMVB data set has been used for multiple correlative studies. It would be interesting to know if molecular analysis or immune staining are available for a subset of patients.
4. The observation of a trend towards improved survival for non-Caucasian Americans is also very interesting and deserves further exploration.

 

 Minor comments:

 The text on page 5 regarding the differences between the therapeutic groups should list the mean survival for the groups and not only the CI. (Also, could a single line of surgery been palliative pleurodesis?)

 Page 6 the n for the primary data set should be changed from 88 to 888.

Is the work clearly and accurately presented and does it cite the current literature?

Yes

If applicable, is the statistical analysis and its interpretation appropriate?

Yes

Are all the source data underlying the results available to ensure full reproducibility?

Yes

Is the study design appropriate and is the work technically sound?

Yes

Are the conclusions drawn adequately supported by the results?

Partly

Are sufficient details of methods and analysis provided to allow replication by others?

Yes

Reviewer Expertise:

Pulmonary Medicine and Thoracic Oncology

I confirm that I have read this submission and believe that I have an appropriate level of expertise to confirm that it is of an acceptable scientific standard.

# Response

**Published:** 2018-12-13

**Authors:** Waqas Amin

**Affiliations:** University of Pittsburgh, USA

1. We agree that our large cohort may not be representative of the general population of such patients and we recognize this weakness in the limitations section. NMVB cohort had very small number of cases that were not surgical candidates for their treatment of disease. We have not analyzed them because of insufficient number for meaningful statistical analysis.
2. The specific treatment information has not collected in NMVB database. However, in future studies we will pull the information of specific treatments and include them in the analysis. Again, this limitation is recognized in the paper.
3. We have highlight the issue related to younger age patients BAP1 mutation status in our introduction section paragraph 2. We would like to point out that we do not have such information in our database as of December 2018.
4. There is a significant number of non-European American participants in the NMVB cohort due to geographic location of resource and its participants. We will further explore racial differences in survival in our future papers.

**Response to Minor Comments:**

Corrected.

# Peer review: Reviewer response for version 1

**DOI:** 10.5256/f1000research.16914.r38743

**Published:** 2018-10-10

**Authors:** Michele Carbone

**Affiliations:** 1 Thoracic Oncology Program, University of Hawaii Cancer Center, Honolulu, HI, USA

This is a well done comprehensive report of analyses performed by a distinguished team of investigators who studied mesothelioma survival in the NMVB.  The information presented is certainly useful to the scientific community. Most NMVB cases are from the States of New York and Pennsylvania where the Authors note there was significant exposure to asbestos.

 Critiques:

1. Mesotheliomas developing in carriers of germline mutations have significant improved survival (see Consensus Report Carbone M., Kanodia S., et al ^1^).The lack of information about genetics is a limiting factor that should be acknowledge and that likely influences the finding that young age is a predictor of prolonged survival as these mesothelioma characteristically occur in young patients.In short this issue should be discussed.
2. The information about asbestos exposure is based on self reported history.This information is often unreliable, as patients who think to have been exposed may not have been exposed and vice versa (asbestos is invisible by the naked eye so it is impossible to be certain whether dust contains or does not contain asbestos fibers, unless the dust is studied at the microscope), as shown for example by comparing results of lung content analyses and self reported history of exposure: see, Carbone M. et al ^2^.The lack of corroborating evidence, such as radiological analyses supporting exposure –about 75% of patients exposed to asbestos develop bilateral plaques, should also be acknowledged.
3. Most recent studies report that presently most pleural mesotheliomas occur in asbestos exposed individuals, and that instead patients with peritoneal mesothelioma rarely report asbestos exposure (for example only 5/64 patients in a recent series by Richard Alexander. Lee M et al ^3^. (What was the proportion of self reported asbestos exposure among patients with pleural or peritoneal mesothelioma?
4. Introduction. Mesothelioma is associated with exposure to professional exposure to asbestos and to environmental exposure to various mineral fibers including asbestos. Clarify this issue, and define what asbestos is. See Baumann F., Buck BJ, et al ^4^; Baumann F et al ^5^.  Moreover, mesotheliomas develops in carriers of germline mutations of BAP1 (Carbone M., Kanodia S., JTO 2016 ^6^, and mutations of BAP1 may increase susceptibility to low doses of asbestos and other mineral fibers (Napolitano A., Pellegrini L., et al ^7^).  These issues are important to understand the reasons of the current ongoing mesothelioma epidemic and also given the different prognosis and survival of mesotheliomas occurring in carriers of BAP1 mutations.

Minor:

 Abstract conclusion last line…..treatment IN PERITONEAL MESOTHELIOMA is associated with improved survival….

 Page 3, introduction, bottom, therapeutic options: ref 15 in the rapidly evolving field of mesothelioma therapy is rather old. Replace or add current reference: the most recent review on this topic is Mutti L., Peikert T., et al ^8^.

Is the work clearly and accurately presented and does it cite the current literature?

Yes

If applicable, is the statistical analysis and its interpretation appropriate?

I cannot comment. A qualified statistician is required.

Are all the source data underlying the results available to ensure full reproducibility?

Yes

Is the study design appropriate and is the work technically sound?

Yes

Are the conclusions drawn adequately supported by the results?

Yes

Are sufficient details of methods and analysis provided to allow replication by others?

Yes

Reviewer Expertise:

mesothelioma and asbestos, environmental carcinogenesis, gene environment interaction, cancer syndromes

I confirm that I have read this submission and believe that I have an appropriate level of expertise to confirm that it is of an acceptable scientific standard, however I have significant reservations, as outlined above.

## References

[1] : Consensus Report of the 2015 Weinman International Conference on Mesothelioma. *J Thorac Oncol*.11(8) : 10.1016/j.jtho.2016.04.028 1246-1262 10.1016/j.jtho.2016.04.028 27453164PMC5551435

[2] : Malignant mesothelioma: facts, myths, and hypotheses. *J Cell Physiol*.2012;227(1) : 10.1002/jcp.22724 44-58 10.1002/jcp.22724 21412769PMC3143206

[3] : Diagnosis and management of patients with malignant peritoneal mesothelioma. *J Gastrointest Oncol*.2016;7(1) : 10.3978/j.issn.2078-6891.2015.134 79-86 10.3978/j.issn.2078-6891.2015.134 26941986PMC4754312

[4] : The Presence of Asbestos in the Natural Environment is Likely Related to Mesothelioma in Young Individuals and Women from Southern Nevada. *J Thorac Oncol*.2015;10(5) : 10.1097/JTO.0000000000000506 731-7 10.1097/JTO.0000000000000506 25668121PMC4406807

[5] : Asbestos is not just asbestos: an unrecognised health hazard. *The Lancet Oncology*.2013;14(7) : 10.1016/S1470-2045(13)70257-2 576-578 10.1016/S1470-2045(13)70257-2 23725699

[6] : High Incidence of Somatic BAP1 alterations in sporadic malignant mesothelioma. *J Thorac Oncol*.2015;10(4) : 10.1097/JTO.0000000000000471 565-76 10.1097/JTO.0000000000000471 25658628PMC4408084

[7] : Minimal asbestos exposure in germline BAP1 heterozygous mice is associated with deregulated inflammatory response and increased risk of mesothelioma. *Oncogene*.2016;35(15) : 10.1038/onc.2015.243 1996-2002 10.1038/onc.2015.243 26119930PMC5018040

[8] : Scientific Advances and New Frontiers in Mesothelioma Therapeutics. *J Thorac Oncol*.2018;13(9) : 10.1016/j.jtho.2018.06.011 1269-1283 10.1016/j.jtho.2018.06.011 29966799PMC6643278

# Response

**Published:** 2018-12-13

**Authors:** Waqas Amin

**Affiliations:** University of Pittsburgh, USA

1. We have highlighted the importance mesotheliomas developing in carriers of germline mutations have significant improved survival. In the limitations, we recognize the fact that we do not have genetic information.
2. We discuss the issue environmental exposure (Natural Occurring Asbestos) and occupational exposure and their association with pleural and peritoneal mesothelioma in introduction section. We recognize that we do not have radiological analysis to support exposure and rely on self-reported data.
3. In our cohort of analysis, the ratio of pleural mesothelioma with asbestos exposure to malignant peritoneal mesothelioma with asbestos exposure is 23:277.
4. Information on various exposures and BAP1 has been added to the introduction

We have updated the citations in the manuscripts and made minor corrections.

# Peer review: Reviewer response for version 1

**DOI:** 10.5256/f1000research.16914.r37581

**Published:** 2018-08-29

**Authors:** Nico van Zandwijk

**Affiliations:** 1 University of Sydney, Sydney, NSW, Australia; 2 Sydney Local Health District, Sydney, NSW, Australia

888 cases (out of 1400 cases) enrolled in the NMVB, representing around 1% of all mesothelioma cases (occurring from 1900 till 2107) in the US, are being used for this prognostic factors study.

 Comparing the distribution of patients in this study with epidemiological studies suggests that over-representation of surgical and peritoneal cases may be present in the series presented. Multivariate analyses in a skewed population may give rise the wrong conclusions, and statistical/epidemiological advice is needed to assure that the conclusions from current analysis are valid.

Is the work clearly and accurately presented and does it cite the current literature?

Yes

If applicable, is the statistical analysis and its interpretation appropriate?

I cannot comment. A qualified statistician is required.

Are all the source data underlying the results available to ensure full reproducibility?

Yes

Is the study design appropriate and is the work technically sound?

Partly

Are the conclusions drawn adequately supported by the results?

Partly

Are sufficient details of methods and analysis provided to allow replication by others?

Yes

Reviewer Expertise:

Thoracic oncology

I confirm that I have read this submission and believe that I have an appropriate level of expertise to confirm that it is of an acceptable scientific standard, however I have significant reservations, as outlined above.

# Response

**Published:** 2018-10-08

**Authors:** Waqas Amin

**Affiliations:** University of Pittsburgh, USA

We would like to thank Reviewer 1 for their thoughtful comments. We would like to point out that, while our paper focuses on 1% of national mesothelioma cases, describing this population is extremely valuable as this group of patients is not a part of SEER and has not been captured by previous research. Findings should be considered in the context of related findings from other populations. While the role of aggressive surgery remains controversial for this groups of patients, few epidemiological studies have evaluated treatment patterns of these patients. The significance of these results, consistent with Reviewer’s comments, is further motivated by the fact that existing studies are flawed by their limited size and inclusion criteria. In the updated version of this paper, we commented (Discussion section) on selection of patients being a potential bias. We also commented on the fact that the treatment of our patients were consistent with ASCO guidelines. Also in the discussion, we pointed out that our conclusions are based on this particular group of patients and more extensive research needs to be implemented on the national and global level to draw more accurate conclusions.

We also acknowledge regression analysis may be insufficient to control for confounding if groups are not largely overlapping. In the case of single exposures or assessing treatment effectiveness, causal inference methods (e.g. propensity score-based methods) may be more appropriate. One of our co-authors (Landsittel) is very familiar with these methods, having served as a PI of a methods contract on the topic (see https://www.pcori.org/research-results/2013/guidance-researchers-optimal-methods-conducting-comparative-effectiveness). However, we did not feel that these methods were entirely applicable since the goals of this project focused on describing a range of risk factor associations, which was still best accomplished through regression. Future analyses could focus on using such methods for more refined questions about a specific exposure.

We have two coauthors, an epidemiologist (Linkov; associate professor of Ob/Gyn and Epidemiology) and a biostatistician (Landsittel; professor of biomedical informatics), who actively participated in the development of this paper, as well as data analysis. Their qualifications, that uniquely correspond to the primary focus of this research, are outlined below. Each has expertise in relevant methods, exposures and disease outcomes, has over 100 publications, and has 15-20 years of experience in research.
